# Effects of intra-accumbal or intra-prefrontal cortex microinjections of adenosine 2A receptor ligands on responses to cocaine reward and seeking in rats

**DOI:** 10.1007/s00213-018-5072-8

**Published:** 2018-11-13

**Authors:** K. Wydra, A. Suder, M. Frankowska, D. O. Borroto Escuela, K. Fuxe, M. Filip

**Affiliations:** 1 Department of Drug Addiction Pharmacology, Institute of Pharmacology Polish Academy of Sciences, 12 Smetna Street, PL-31-343 Kraków, Poland; 2 Department of Neuroscience, Karolinska Institutet, Stockholm, Sweden

**Keywords:** Adenosine A_2A_ receptor ligands, Cocaine self-administration, Cocaine seeking, Local microinjection, Nucleus accumbens, Prefrontal cortex, Rats

## Abstract

**Rationale and objectives:**

Many studies indicated that adenosine via its A_2A_ receptors influences the behavioral effects of cocaine by modulating dopamine neurotransmission. The hypothesis was tested that A_2A_ receptors in the nucleus accumbens (NAc) or the prefrontral cortex (PFc) may modulate cocaine reward and/or cocaine seeking behavior in rats.

**Methods:**

The effects of local bilateral microinjections of the selective A_2A_ receptor agonist CGS 21680 or the A_2A_ receptor antagonists KW 6002 and SCH 58261 were investigated on cocaine self-administration on reinstatement of cocaine seeking.

**Results:**

The intra-NAc shell, but not intra-infralimbic PFc, administration of CGS 21680 significantly reduced the number of active lever presses and the number of cocaine (0.25 mg/kg) infusions. However, tonic activation of A_2A_ receptors located in the NAc or PFc did not play a role in modulating the rewarding actions of cocaine since neither KW 6002 nor SCH 58261 microinjections altered the cocaine (0.5 mg/kg) infusions. The intra-NAc but not intra-PFc microinjections of CGS 21680 dose- dependently attenuated the reinstatement of active lever presses induced by cocaine (10 mg/kg, i.p.) and the drug-associated combined conditioned stimuli using the subthreshold dose of cocaine (2.5 mg/kg, i.p.). On the other hand, the intra-NAc pretreatment with SCH 58261, but not with KW 6002, given alone evoked reinstatement of cocaine seeking behavior.

**Conclusion:**

The results strongly support the involvement of accumbal shell A_2A_ receptors as a target, the activation of which exerts an inhibitory control over cocaine reward and cocaine seeking.

## Introduction

Substance-use disorder (drug addiction) is a chronic relapsing disorder characterized by compulsive drug intake and drug seeking, loss of control over drug intake, and a persistent craving for the drug. The neuronal basis of cocaine addiction includes activation of the mesocorticolimbic circuitry with changes in dopamine (DA) and glutamate neurotransmissions (Arbuthnott et al. 1970; Kalivas 2009; Koob 2009).

The meso-limbic DA system (Fuxe 1965) was linked mainly to compulsive drug use. In fact, drugs of abuse increase release of DA in the shell subregion of the nucleus accumbens (NAc) (Di Chiara 2002). The glutamate system was linked mainly to relapse after drug seeking and the circuitry included a glutamate pathway from the prefrontal cortex (PFc) to the NAc (McFarland and Kalivas 2001). In fact, administration of cocaine into the PFc restored seeking behavior (Park et al. 2002).

A few years ago, it was shown that adenosine may influence DA and glutamatergic neurotransmission in several brain structures including the NAc and PFc region (Fuxe et al. 2007; Fuxe et al. 2008). Adenosine acts through A_1_, A_2A_, A_2B_, and A_3_ receptors, among which A_2A_ receptors are highly enriched in certain brain areas including striatum while less in other brain regions such as the cerebral cortex (Schiffmann et al. 2007; Sihver et al. 2009). In neurochemical studies it was showed that local or systemic activation of A_2A_ receptors in dorsal and ventral striatum enhanced the release of DA and glutamate (Golembiowska and Zylewska 1997; Popoli et al. 1995; Quarta et al. 2004; Tanganelli et al. 2004) while stimulation of A_2A_ receptors in medial PFc reduced striatal DA release (Acquas et al. 1999). On the other hand, the DA or glutamate release under control over A_2A_ receptors is not unequivocally established as either reduction (Quarta et al. 2004), increase (Harper et al. 2006), or no effect (Quarta et al. 2004; Solinas et al. 2002) was described following the local receptor blockade in the rat ventral and/or dorsal striatum.

A_2A_ receptors have been also linked to substance use disorders (Filip et al. 2012). Regarding cocaine addiction previous pharmacological studies with A_2A_ receptor ligands using systemic drug administration indicated that A_2A_ receptors play a significant role in the drug-induced reward, motivation, and seeking (Bachtell and Self 2009; Filip et al. 2006, 2012; Knapp et al. 2001; Wydra et al. 2015a, b). Moreover, O'Neill et al. (2012) demonstrated in rats that A_2A_ receptors in the NAc core bi-directionally alter cocaine seeking with the agonism-evoked reduction and the antagonism-provoked reinstatement. The pharmacological findings with A_2A_ receptor antagonists collaborate with genetic studies using receptor striatal-specific knockdown where the enhancement of cocaine locomotion was observed (Shen et al. 2008), while A_2A_ receptor knockout (Chen et al. 2000, 2003; Soria et al. 2006) or a forebrain–specific knockdown of A_2A_ receptors (Shen et al. 2008) decreased cocaine reward or locomotion, respectively. Despite that cocaine self-administration and relapse behaviors are the goal-directed behavior, being controlled by habit (cf. Root et al. 2009), many recent papers with using pharmacological or genetic tools strongly demonstrated the contribution of striatal A_2A_ receptors to goal-directed behavior and the effort-related behaviors (Chen 2014; Correa et al. 2016; Li et al. 2016; Mingote et al. 2008; Nunes et al. 2013; Pardo et al. 2012, 2013; Pereira et al. 2011; Randall et al. 2012; Yu et al. 2009) in different preclinical models.

To further investigate the role of different brain A_2A_ receptor population in the current study cocaine self-administration and cocaine extinction/reinstatement protocols as well as local microinjection procedures were employed to test the hypothesis that A_2A_ receptors in NAc and PFc may modulate the behavioral actions of cocaine. The effects of the selective A_2A_ receptor antagonists KW 6002 (K_i_ = 2.2 nM) and SCH 58261 (K_i_ = 1.3 nM) (Zocchi et al. 1996) and the selective receptor agonist CGS 21680 (K_i_ = 17 nM) were studied. KW 6002 appears to block with a similar potency A_2A_ protomers belonging to A_2A_ homoreceptor and A_2A_ heteroreceptor complexes as far as A_2A_-D_2_ and A_2A_-A_2A_ complexes are concerned (Orru et al. 2011a). SCH 58261 has not been characterized in this respect. The A_2A_ antagonists and the A_2A_ agonist were administered directly into NAc shell or infralimbic PFc during cocaine self-administration and during cocaine or cocaine-associated conditional stimulus (cue) seeking behavior in rats.

## Experimental procedures

### Animals

Male Wistar rats (derived from the licensed animal breeder Charles River, Sulzfeld, Germany), weighing between 260 and 310 g at the beginning of the experiment, were used. The animals were housed individually in standard plastic rodent cages (39 cm × 28 cm × 28 cm) at a room temperature of 21 ± 1 °C and at a 40 ± 5% humidity with a 12-h light–dark cycle (lights on at 6:00 a.m.). Animals had free access to food (VRF1 pellets, UK) and water (except for the initial training session (see below). All experiments were carried out in accordance with EU directive 2010/63/EU and with approval of the Local Ethics Commission.

### Drugs

Cocaine (3β-hydroxy-1α*H*,5α*H*-tropane-2β-carboxylic acid methyl ester benzoate hydrochloride; Sigma-Aldrich; USA), KW 6002 (8-[(1*E*)-2-(2(3,4-dimethoxyphenyl)ethenyl]-1,3-diethyl-3,7-dihydro-7-methyl-1*H*-purine-2,6-dione; Tocris, UK), SCH 58261 (2-(2-furanyl)-7-(2-phenylethyl)-7*H*-pyrazolo[4,3-*e*][1,2,4]triazolo[1,5-*c*]pyrimidin-5-amine; Tocris, UK), and CGS 21680 (4-[2-[[6-amino-9-(*N*-ethyl-*b*-d-ribofuranuronamidosyl)-9*H*-purin-2-yl]amino]ethyl]benzene-propanoic acid hydrochloride; Tocris, UK). Cocaine and CGS 21680 were dissolved in 0.9% NaCl; KW 6002 was dissolved in a mixture (1:1:8) of dimethyl sulfoxide (DMSO, Sigma-Aldrich, USA), Tween®80 (Sigma-Aldrich, USA), and 0.9% NaCl, while SCH 58261 was dissolved in 1% DMSO. CGS 21680, KW 6002, and SCH 58261 were administrated intra-NAc or intra-PFc immediately before 2-h self-administration session in a volume of 0.2 μl/min per side. Cocaine was administered i.v. in a volume of 0.1 ml per infusion or i.p in a volume 1 ml/kg. Doses of A_2A_ receptor ligands were established based on our preliminary data (Filip et al. 2017; Wydra et al. 2017) and on previous behavioral studies with using microinjection procedures that show efficacy of the A_2A_ receptor agonist in doses 2.5–24 ng that dose-dependently decreased active lever presses for cocaine seeking (O'Neill et al. 2012), decreased home cage activity (Sardi et al. 2018) or disrupted performance of an instrumental task with high work demands together with increased extracellular GABA levels in the ventral pallidum (Mingote et al. 2008). The doses of KW 6002 and SCH 58261 were chosen based on the intra-accumbal dose range of another A_2A_ receptor antagonist MSX-3 that displays similar potency to KW 6002 and SCH 58261 for the receptors in context of radioligand binding and cAMP assays (Yang et al. 2007). In fact, intra-NAc administration of MSX-3 increased cocaine seeking (5–20 μg/side; O'Neill et al. 2012) or enhanced locomotor activity in an open field after intra-NAc (5 μg; Nagel et al. 2003), or intra-striatal (9 μg; Hauber et al. 1998) MSX-3 administration. The recent paper indicated that SCH 58261 in a dose range of 20–40 μg/side repeatedly injected intracerebroventricularly exerted inhibitory effect on astrocytes activation and prevented the spatial memory impairment in rats (Akbari et al. 2018).

### Self-administration procedures

#### Intravenous catheter implantation

After 18-h water deprivation, animals were trained for 5 days to press a lever for 2-h daily in standard operant chambers (Med-Associates, St. Albans, GA, USA) under a fixed ratio (FR) schedule 1–5 of water reinforcement. Two days after lever pressing training and free access to food and water, rats were anesthetized with intramuscular injection of ketamine hydrochloride (75 mg/kg, i.m; Biowet, Poland) and xylazine (5 mg/kg, i.m; Biowet, Poland) and implanted with a silastic catheter in the external right jugular vein, as described previously (Filip et al. 2007). Catheters were flushed daily with 0.2 ml of saline solution containing cephazolin (100 mg/ml; Biochemie GmbH, Austria) and heparin (100 U/ml; Biochemie GmbH, Austria) to prevent catheter non-patency as a result of blood clotting.

#### Guide cannulae implantation and microinjection procedure

Immediately after the catheter implantation, rats were stereotaxically implanted with stainless steel bilateral guide cannula (22-gauge, 10 mm long; Plastic One, USA). A guide cannula was implanted stereotaxically into the NAc at the following coordinates from the Bregma: [anteroposterior (AP) = 1.7 mm; mediolateral (ML) = ± 0.75 mm and dorsoventral (DV) = −6 mm] and the PFc [AP = 2.7 mm; ML = ±0.75 mm and DV = −3 mm] according to the rat brain atlas (Paxinos and Watson 1998). The coordinated were chosen on the basis that the NAc shell is engaged in the control of cocaine reward (Di Chiara et al. 2004; Marie et al. 2012; Müller Ewald et al. 2018) while medial parts of the NAc (shell and medial core) are linked with cocaine seeking (Bachtell and Self 2009; Schmidt et al. 2006). A main efferent projection from the infralimbic PFc is to the NAC shell and both these brain structures are recruited by the extinction learning to control cocaine seeking (Peters et al. 2008). The guide cannula was affixed to the skull with two miniature stainless steel screws (Agnatho’s, Sweden) and dental acrylic cement. After the surgery, all animals had a 6–8-day recovery period.

The microinjection unit was organized from a polyethylene tubing (OD 0.023 mm, ID 0.041 mm, Plastic One, USA) connected to two 1-ml Hamilton syringes at one end and a bilateral injection cannula (28-gauge, 12-mm length; Plastic One, USA) at the other end. On the day of the test, a bilateral internal cannula was inserted into the guide cannula after obturator removal. The microinjection volume of 0.2 μl was delivered bilaterally over 1 min by the syringe pump drive (BAS, USA), operated with a programmable controller (Bee Hive Controller; BAS, USA). A diffusion time of 1 min was allowed before the removal of the injection cannula and replacement of the obturator. The rat received three-four microinjections into the NAc or PFc.

#### Maintenance of cocaine self-administration

Following recovery, the animals were given access to cocaine during 2-h daily sessions performed 6 days/week. The house light was illuminated throughout each session. Each press on the “active” lever (FR 5 schedule of reinforcement) resulted in a 5-s infusion of cocaine (0.5 mg/kg per 0.1 ml) and a 5-s presentation of a stimulus complex (activation of the white stimulus light directly above the “active” lever and the tone generator, 2000 Hz; 15 dB above ambient noise levels). Following each injection, there was a 20-s time-out period during which responding was recorded, but had no programmed consequences. Presses on the “inactive” lever were recorded, but not reinforced. Acquisition of the conditioned operant response lasted a minimum of 9 days until subjects met a stable average of three consecutive days and a standard deviation within those days of < 10% of the average (Filip et al. 2007).

After the acquisition criterion (see above) was met, separate groups of rats (N = 8 rats/group) were used to complete a cocaine (0.25–0.5 mg/kg/infusion) dose–response curve.

Later following stabilization of responding, the rats underwent microinjection procedures (see above). Local microinjections of CGS 21680 (1–10 ng/side), KW 6002 (1–5 μg/side), or SCH 58261 (1–2.5 μg/side) were given to separate groups of animals.

**Fig. 1:**
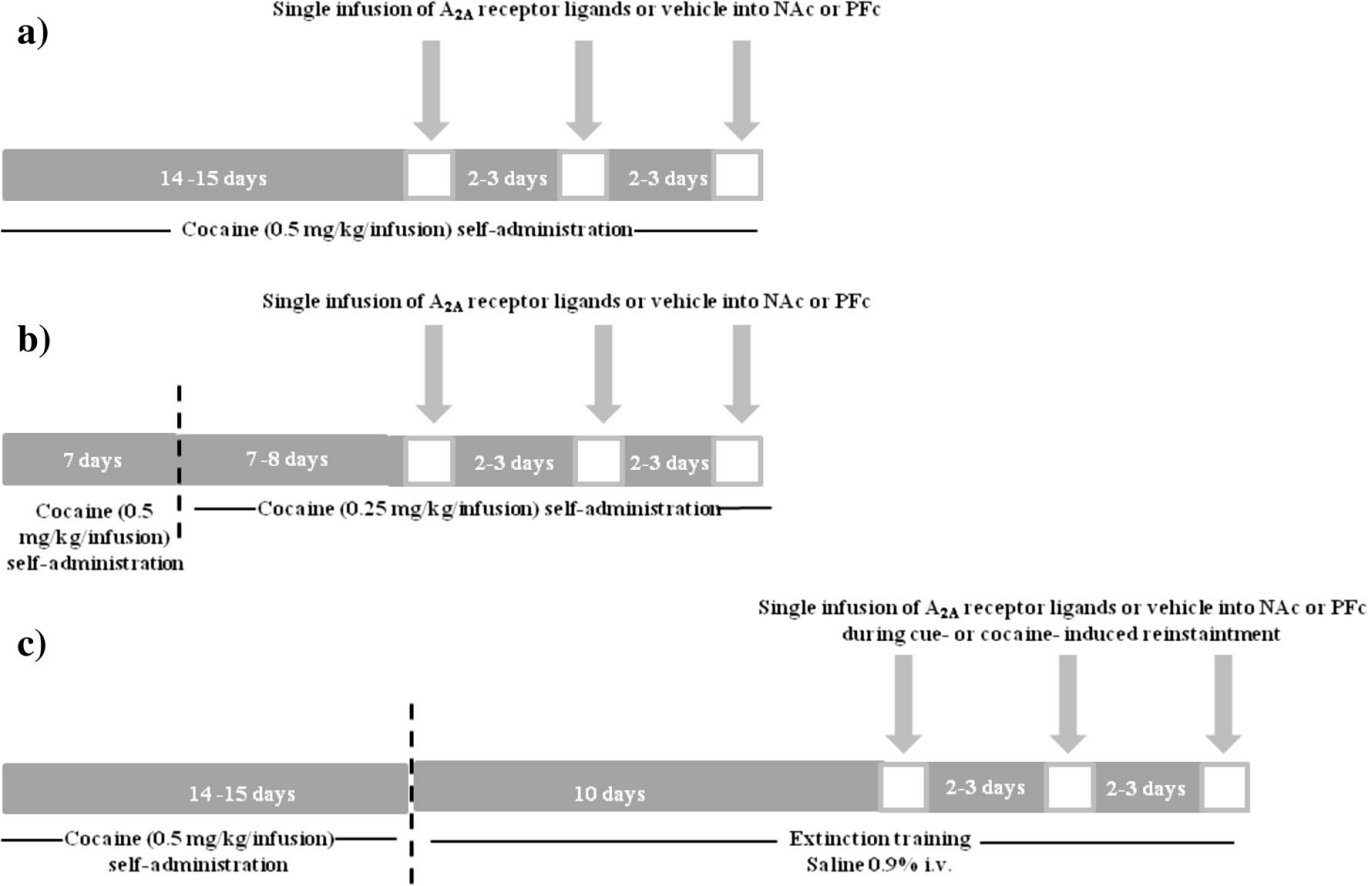
Experimental design of the study. Schematic diagrams show cocaine (0.5 mg/kg/infusion) self-administration (**a**), cocaine (0.25 mg/kg/infusion) self-administration (**b**), and extinction training/reinstatement (**c**) procedures with intracranial microinjections of A_2A_ receptor ligands

A maximum of three test sessions was performed on each rat group, separated by at least two to three baseline days of cocaine self-administration (Fig. 1a, b). The order of injections was counterbalanced according to a Latin square design.

#### Extinction/reinstatement

During extinction sessions, rats had 2-h daily training sessions with no delivery of cocaine or the presentation of the conditioned stimulus. Once they reached the extinction criteria (a minimum of 10 extinction days with the responding on the “active” lever below ~ 10% of the active lever presses observed during at least the three last days of the maintenance phase), animals were tested for response reinstatement induced by a non-contingent presentation of cocaine (2.5 or 10 mg/kg, i.p.), a conditioned stimulus cue (tone + light previously paired with cocaine self-administration) alone or with the subthreshold dose (2.5 mg/kg) of cocaine. Additionally, based on our previous data with systemic drug injections showing that A_2A_ receptor antagonists can reinstate cocaine seeking behavior (Wydra et al. 2015b), we tested KW 6002 and SCH 58261 alone for response reinstatement.

During the reinstatement tests (2-h sessions), active lever presses on the FR 5 schedule resulted in intravenous injection of saline only. Drug combination was given in a randomized order in maximum of three-four reinstatement tests. Each rat underwent only one type of the reinstatement procedure (above) (Fig. 1c). The order of injections was counterbalanced according to a Latin square design, and the test sessions were separated by at least two to three baseline days of the extinction sessions.

### Locomotor activity procedures

#### Surgery

Rats were stereotaxically implanted with stainless steel bilateral guide cannulae as described above.

### Measurement of locomotor activity

Following recovery period (6–8 days) the locomotor activity of non-habituated rats was recorded for each animal. Locomotor activity was measured in Opto-Varimex cages framed by a 15 × 15 array of photocell beams located 3 cm above the floor surface (Columbus Instruments, Columbus, USA). Photobeam breaks resulted in a measure of horizontal activity defined as a distance traveled (expressed in cm). The animals were placed individually in locomotor activity cages for 2-h, their locomotion was recorded and analyzed using Auto-track software (Columbus Instruments, USA). Before the locomotor activity was recorded, rats (N = 8 rats per/group) were microinjected (intra-NAc or intra-PFc) with KW 6002 (1, 2.5, 5 μg/side), SCH 58261 (1, 2.5, 5 μg/side), or with CGS 21680 (1, 2.5, 10 ng/side) and transferred to the experimental cages.

### Histology

**Fig. 2:**
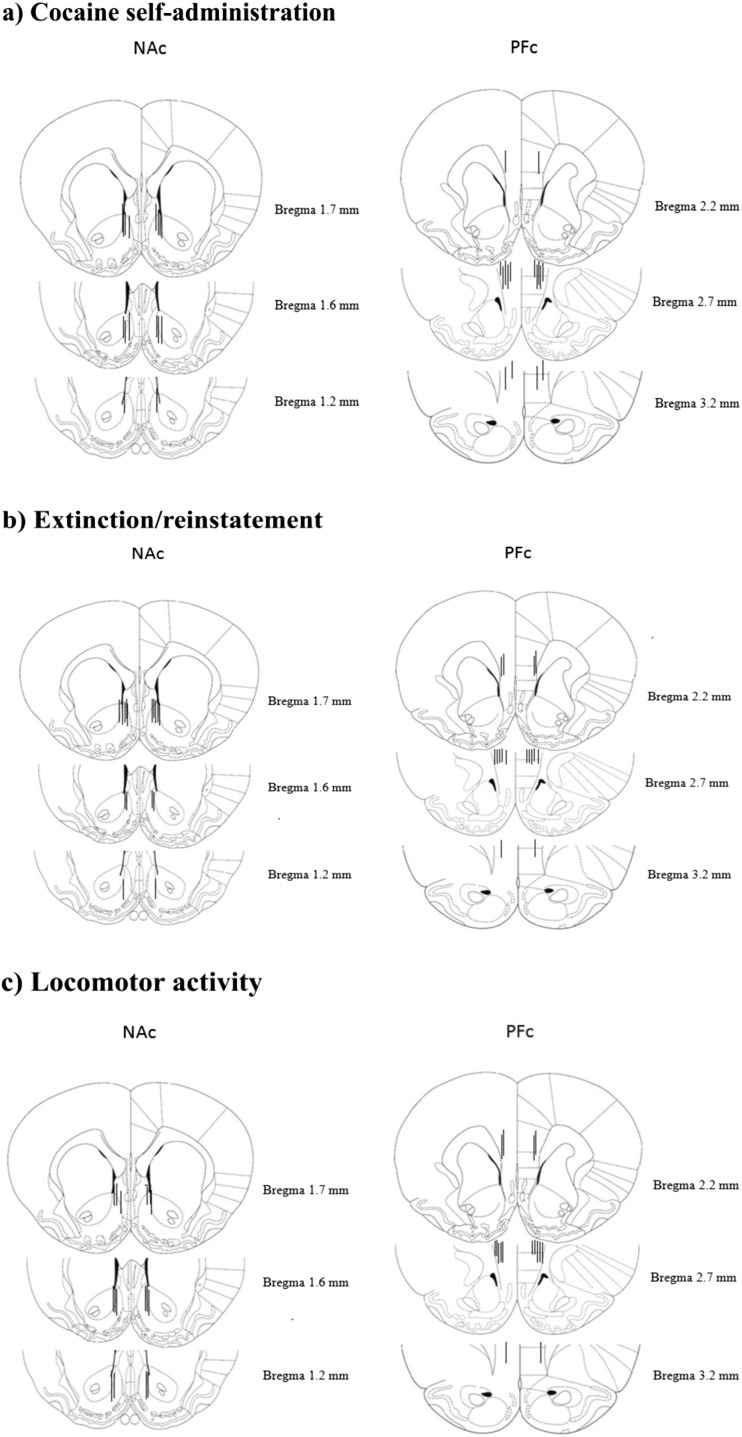
Histological verification of microinjection representative probe placements in the NAc (left panels) and the PFc (right panels) of rats that underwent cocaine self-administration (**a**), extinction/ reinstatement tests (**b**), and locomotor activity (**c**). Plates are taken from rat brain atlas Paxinos and Watson (1998) and the black line represent right placement of probes. Due to the large number of animals utilized for studies, bilateral placements are shown for only a subset of the experimental pool

Immediately after the completion of the experiments, rats were overdosed with sodium pentobarbital (morbital; 133.3 mg/ml; i.p.; Biowet, Poland) and the brains were removed and stored in a 4% formalin (POCH, Poland) solution for at least 3 days before the sectioning Brains were cut into 12-μm sections on a cryostat, mounted on gel-coated glass slides. The brain sections were defatted, stained with cresyl violet, cleared with xylene and placed under coverslips. The placement of microinjection probes were verified using a light microscope. There was no necrosis distal to the track upon histological examination of sections. Only data from rats with correctly placed probes within the NAc and the PFc according to previously established guidelines were included for statistical analyses (Fig. 2).

### Statistical analysis

The obtained results are presented as the means ± SEM. The number of responses on the active and inactive lever and a number of cocaine infusions were analyzed using a factorial analysis of variance (ANOVA), followed by post hoc Dunnett’s or Newman-Keuls test.

Locomotor activity data are expressed as the mean total horizontal distance traveled in cm for a 2-h test session. Comparisons between groups were carried out by one-way ANOVA, followed by the Dunnett’s test.

## Results

### Cocaine reward

The rats showed stable responding to levers during the last 3 cocaine (0.5 mg/kg/infusion) self-administration maintenance sessions with an acquisition criterion requiring that the rate of active lever presses varied by less than 10%. The animals used for the intra-NAc and intra-PFc microinjection studies had 26–30 or 22–30 self-administrated infusions of cocaine with the daily mean cocaine intake amounting to 12–15 mg/kg or 11–15 mg/kg, respectively. The animals responded significantly more frequently to the active lever vs the inactive lever (*p* < 0.05).

When the dose of cocaine was reduced to 0.25 mg/kg/infusion the animals had 39–50 or 33–53 self-administrated infusions of cocaine with the daily mean cocaine intake amounting to 9.75–12.5 mg/kg or 8.25–13.25 mg/kg for the intra-NAc or the intra-PFc microinjection studies, respectively. The animals responded significantly more frequently to the active lever vs the inactive lever (*p* < 0.05).

### Intra-NAc effects of A_2A_ receptor ligands on cocaine (0.25 mg/kg/infusion) self-administration

**Fig. 3:**
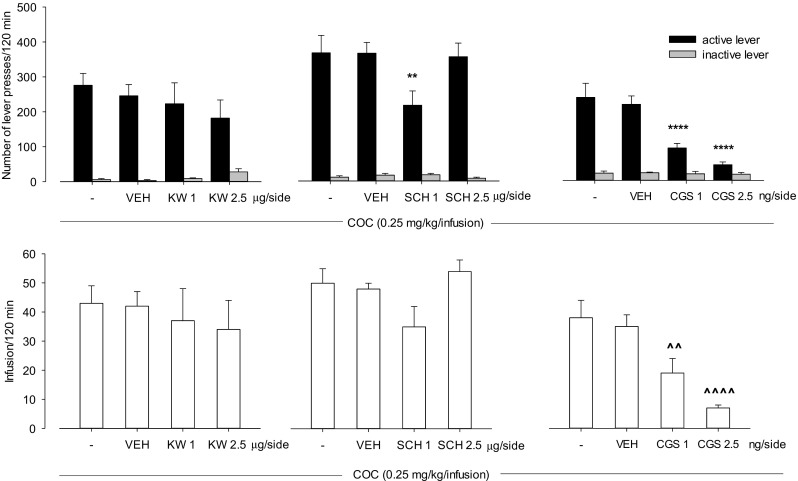
Intra-NAc effects of A_2A_ receptor antagonists KW 6002 (KW; 1–2.5 μg/side), SCH 58261 (SCH; 1–2.5 μg/side), and CGS 21680 (CGS; 1–2.5 ng/side) on cocaine (COC; 0.25 mg/kg/infusion) self-administration in rats. The number of active and inactive lever presses (upper panels) and the number of cocaine infusions (lower panels) are shown as the mean (±SEM). **p < 0.01, ****p < 0.0001 vs vehicle (VEH) (Newman-Keuls test); ^^^^*p* < 0.01, ^^^^^^*p* < 0.0001 vs (VEH) (Dunnett’s test). *N* = 6–8 rats/group

Two-way ANOVA indicated that KW 6002 (1–2.5 μg/side; *F*(2,30) = 0.48, *P* = 0.63) did not change the number of active and inactive lever presses. However, SCH 58261 at a dose 1 μg/side decreased the number of active lever presses (*F*(2,30) = 5.33, *P* < 0.01) (Fig. 3, upper panels). One-way ANOVA showed that KW 6002 (*F*(2,15) = 0.21, *P* = 0.81) and SCH 58261 (*F*(2,15) = 4.04, *P* = 0.04) did not alter cocaine reinforcements (Fig. 3, lower panels).

Two-way ANOVA indicated a significant effect of CGS 21680 (1–2.5 ng/side) on pretreatment × lever interaction (*F*(2,30) = 25.2, *P* < 0.0001). The post hoc Newman-Keuls test revealed that CGS 21680 in doses of 1 and 2.5 ng/side reduced the number of active lever presses by 58% (*p* < 0.0001) and 79% (*p* < 0.0001), respectively, without any changes in the number of inactive lever presses (Fig. 3, upper panel).

One-way ANOVA showed that CGS 21680 (1–2.5 ng/side) altered cocaine reinforcements (*F*(2,15) = 15.61, *P* < 0.001). The post hoc Dunnett’s test revealed that CGS 21680 in doses 1 and 2.5 ng/side reduced cocaine reward by 45% (*p* < 0.01) and 80% (*p* < 0.001), respectively (Fig. 3, lower panel).

### Intra-NAc effects of A_2A_ receptor ligands on cocaine (0.5 mg/kg/infusion) self-administration

**Fig. 4:**
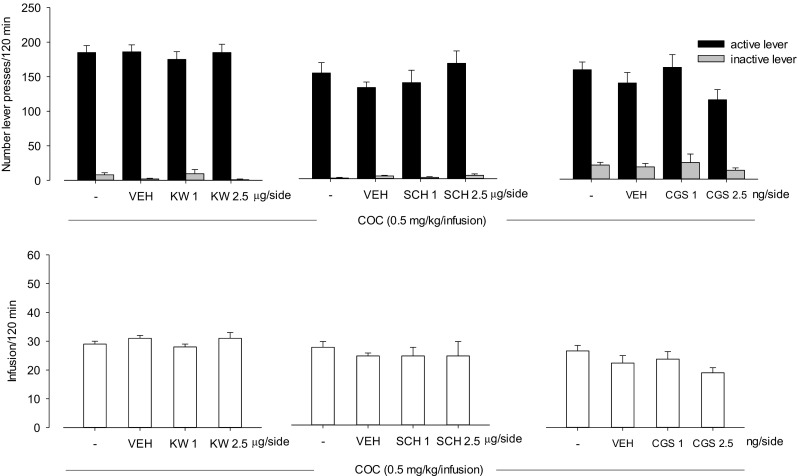
Intra-NAc effects of A_2A_ receptor antagonists KW 6002 (KW; 1–2.5 μg/side), SCH 58261 (SCH; 1–2.5 μg/side), and CGS 21680 (CGS; 1–2.5 ng/side) on cocaine (COC; 0.5 mg/kg/infusion) self-administration in rats. The number of active and inactive lever presses (upper panels) and the number of cocaine infusions (lower panels) are shown as the mean (±SEM). VEH—vehicle. *N* = 6–8 rats/group

Two-way ANOVA revealed that KW 6002 (1–2.5 μg/side; F(2,42) = 0.57, *P* = 0.57), SCH 58261 (1–2.5 μg/side; *F*(2,36) = 1.10, *P* = 0.34) and CGS 21680 (1–2.5 ng/side; *F*(2,42) = 0.78, *P* = 0.46) did not change the number of active and inactive lever presses (Fig. 4, upper panels).

Similarly, one-way ANOVA showed that KW 6002 (*F*(2,21) = 1.11, *P* = 0.35), SCH 58261 (*F*(2,18) = 0.01, *P* = 0.99), and CGS 21680 (*F*(2,21) = 0.86, *P* = 0.44) did not alter cocaine reinforcements (Fig. 4, lower panel).

### Intra-PFc effects of A_2A_ receptor ligands on cocaine (0.25 mg/kg/infusion) self-administration

Two-way ANOVA revealed that KW 6002 (1–2.5 μg/side; *F*(2,28) = 0.65, *P* = 0.53), SCH 58261 (1–2.5 μg/side; *F*(2,24) = 0.21, *P* = 0.81), and CGS 21680 (1–2.5 ng/side; *F*(2,42) = 0.22, *P* = 0.80) did not alter the number of active and inactive levers presses. Similarly, one-way ANOVA showed that KW 6002 (*F*(2,14) = 0.54, *P* = 0.59), SCH 58261 (*F*(2,12) = 0.67, *P* = 0.53), and CGS 21680 (*F*(2,21) = 0.30, *P* = 0.74) did not modulate the number of cocaine reinforcements (Table 1).

**Table 1:** Intra-PFc effects of KW 6002, SCH 58261, and CGS 21680 on cocaine self-administration. The number of lever presses and the mean of cocaine infusions are shown as the mean (± SEM) from 5 to 8 rats/group

Treatment and dose	Cocaine (mg/kg/infusion) self-administration	Number of active lever presses	Number of inactive lever presses	Number of cocaine infusions
KW 6002 0 μg/side	0.25	306 ± 16	3 ± 0.69	53 ± 2.4
KW 6002 1 μg/side	0.25	312 ± 13	9 ± 1.59	54 ± 4.3
KW 6002 2.5 μg/side	0.25	287 ± 12	5 ± 1.33	47 ± 7.1
KW 6002 0 μg/side	0.5	232 ± 34	0 ± 0	28 ± 4.2
KW 6002 1 μg/side	0.5	225 ± 25	10 ± 7.5	33 ± 2.8
KW 6002 2.5 μg/side	0.5	237 ± 8.2	10 ± 5.3	31 ± 1.2
SCH 58261 0 μg/side	0.25	328 ± 59	10 ± 5.0	53 ± 9.9
SCH 58261 1 μg/side	0.25	306 ± 92	9 ± 2.7	35 ± 10
SCH 58261 2.5 μg/side	0.25	257 ± 68	11 ± 4.9	41 ± 11
SCH 58261 0 μg/side	0.5	154 ± 24	6 ± 1.8	24 ± 2.6
SCH 58261 1 μg/side	0.5	147 ± 12	2 ± 0.8	25 ± 1.9
SCH 58261 2.5 μg/side	0.5	134 ± 12	1 ± 0.5	22 ± 1.6
CGS 21680 0 ng/side	0.25	198 ± 12	9 ± 3.8	33 ± 1.5
CGS 21680 5 ng/side	0.25	196 ± 6.9	6 ± 2.4	31 ± 1.9
CGS 21680 10 ng/side	0.25	191 ± 2.4	8 ± 3.4	31 ± 1.5
CGS 21680 0 ng/side	0.5	165 ± 14	8 ± 2.9	26 ± 0.9
CGS 21680 1 ng/side	0.5	163 ± 14	6 ± 3.1	27 ± 2.5
CGS 21680 2.5 ng/side	0.5	156 ± 8.2	6 ± 1.7	26 ± 1.9

### Intra-PFc effects of A_2A_ receptor ligands on cocaine (0.5 mg/kg/infusion) self-administration

Two-way ANOVA revealed that KW 6002 (1–2.5 μg/side; *F*(2,30) = 0.27, *P* = 0.76), SCH 58261 (1–2.5 μg/side; *F*(2,30) = 0.24, *P* = 0.79), and CGS 21680 (5–10 ng/side; *F*(2,42) = 0.11, *P* = 0.89) did not change the number of active and inactive lever presses. Similarly, one-way ANOVA showed that KW 6002 (*F*(3,20) = 0.89, *P* = 0.46), SCH 58261 (*F*(2,15) = 0.73, *P* = 0.50), and CGS 21680 (*F*(2,21) = 0.09, *P* = 0.91) did not alter cocaine reinforcements (Table 1).

### Cocaine seeking and relapse

After about 12 experimental sessions, rats met the criterion of a stable cocaine (0.5 mg/kg/infusion) self-administration. During maintenance phase, the mean numbers of responses emitted at active lever ranged from 187 ± 16, while the number of inactive lever presses did not exceed 15. After about 12 days of extinction trials during which active lever presses resulted in an i.v. delivery of saline without presentation of a conditioned stimulus (cue), the rats were tested for the response reinstatement induced by cocaine (2.5–10 mg/kg, i.p.), or by presentation of the conditioned stimulus alone and in the presence of a subthreshold dose of cocaine (2.5 mg/kg, i.p.).

**Fig. 5:**
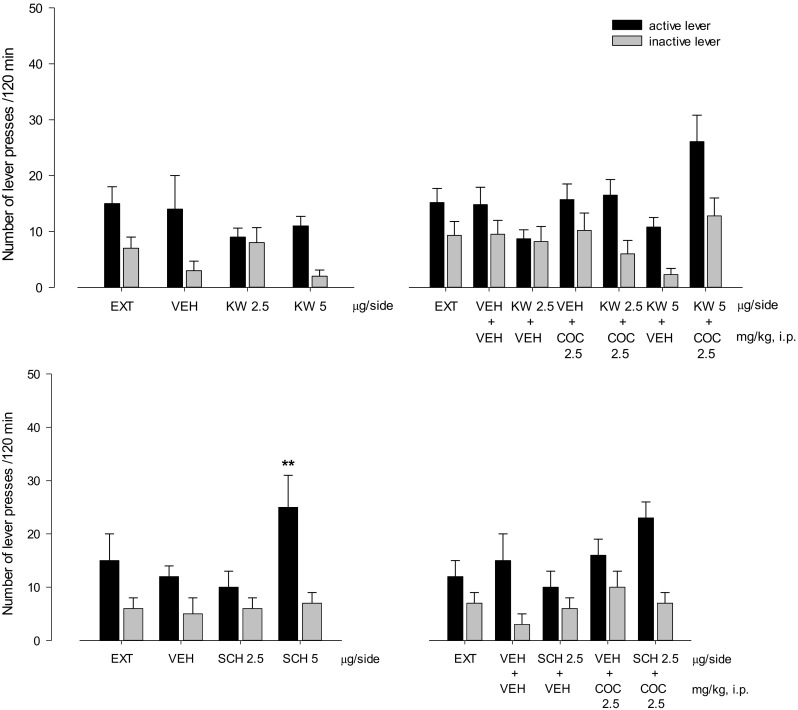
Intra-NAc effects of the A_2A_ receptor antagonists KW 6002 (KW; 2.5–5 μg/side) and SCH 58261 (SCH; 2.5–5 μg/side) alone (left panels) or in combination with cocaine (COC; 2.5 mg/kg, i.p; right panels) on the reinstatement of cocaine seeking in rats. The number of active and inactive lever presses is shown as mean (± SEM). VEH—vehicle, EXT—extinction training last session. *N* = 6 rats/group

**Fig. 6:**
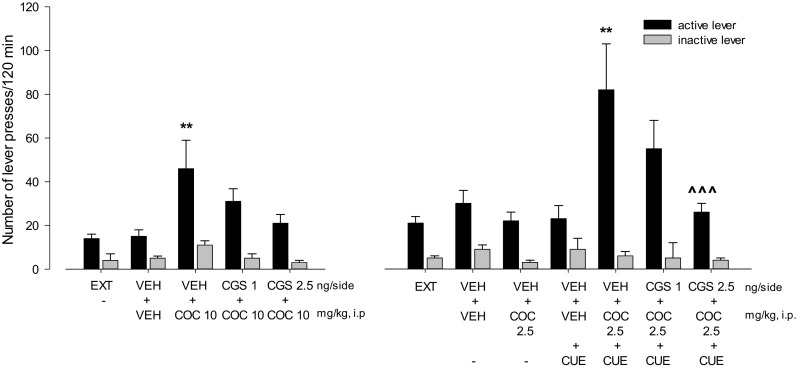
Intra-NAc effects of the A_2A_ receptor agonist CGS 21680 (CGS; 1–2.5 ng/side) on cocaine (COC; 10 mg/kg; left panel) or on the cue (CUE; light and tone previously associated with cocaine self-administration) plus the subthreshold dose of cocaine (COC; 2.5 mg/kg; right panel) on the reinstatement of cocaine seeking behaviors in rats. The number of active and inactive lever presses is shown as mean (± SEM). VEH—vehicle, EXT—extinction training last session.**p < 0.01 vs VEH + VEH; ^^^^^*p* < 0.001 vs VEH + COC 2.5 + CUE (Newman-Keuls test). *N* = 7 rats/group

**Fig. 7:**
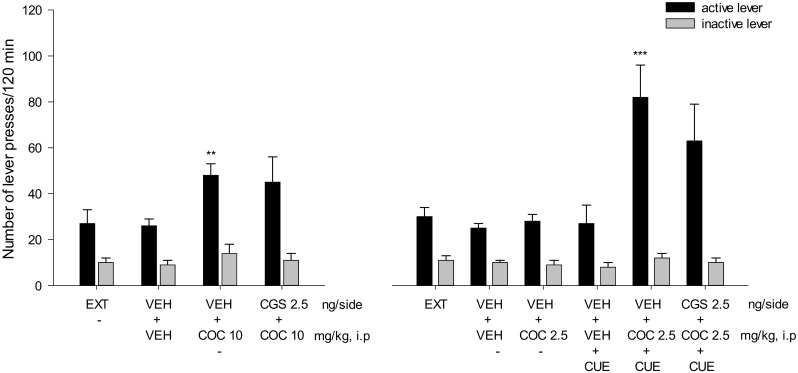
Intra-PFc effects of the A_2A_ receptor agonist CGS 21680 (CGS; 1-2.5 ng/side) on cocaine (COC; 10 mg/kg; left panel) or on the cue (CUE; light and tone previously associated with cocaine self-administration) plus the subthreshold dose of cocaine (2.5 mg/kg; right panel) on the reinstatement of cocaine seeking behaviors in rats. The number of active and inactive lever presses is shown as the mean (± SEM). VEH—vehicle, EXT—extinction training last session.**p < 0.01, ***p < 0.001 vs VEH + VEH (Newman-Keuls test). *N* = 7 rats/group

Cocaine (2.5–10 mg/kg) significantly altered the number of active lever presses (*F*(2,36) = 3.25, *P* < 0.05) without a change in the inactive lever presses. A significant effect was observed following cocaine at 10 mg/kg (*p* < 0.001), but not at 2.5 mg/kg (Figs. 5, 6, and 7).

The animals showed no response to the conditioned stimulus (*F*(1,20) = 0.28, *P* = 0.59) for active and inactive lever presses (Fig. 6, right panel). But, when the conditioned stimulus was combined with a subthreshold dose of cocaine (2.5 mg/kg, i.p.), the significant increase in the number of active lever presses (*F*(1,21) = 5.73, *P* < 0.03) without a change in the inactive lever presses was observed (Fig. 6, right panel).

### Intra-NAc effects of the A_2A_ receptor ligands on cocaine seeking behavior

Based on our previous findings that systemic administration of A_2A_ receptor antagonists induce reinstatement of cocaine seeking (Wydra et al. 2015b), we investigated the effects of local microinjections of the A_2A_ receptor antagonists given alone on the cocaine seeking. Two-way ANOVA for treatment × lever interaction did not indicate a significant effect of KW 6002 (2.5–5 μg/side) on cocaine seeking behavior (*F*(2,30) = 1.34, *P* = 0.28) (Fig. 5, left upper panel).

Two-way ANOVA for treatment × lever interaction indicated a significant effect of SCH 58261 (2.5–5 μg/side) on cocaine seeking behavior (*F*(2,30) = 3.11, *P* < 0.05). The post hoc Newman-Keuls test revealed that SCH 58261 at a dose of 5 μg/side significantly (*p* < 0.01) increased the number of active lever presses without any change in the number of inactive lever presses (Fig. 5, left bottom panel).

Neither KW 6002 (2.5 and 5 μg/side; *F*(1,40) = 1.69, *P* = 0.20) nor SCH 58261 (2.5 μg/side; *F*(1,40) = 3.18, *P* = 0.08) altered significantly the effects of the subthreshold cocaine (2.5 mg/kg; i.p.) dose as shown by three-way ANOVA for pretreatment × treatment × level interaction (Fig. 5, right upper and bottom panels).

Two-way ANOVA for treatment × lever interaction showed a significant effect of CGS 21680 (1–2.5 ng/side) on cocaine (10 mg/kg)-induced reinstatement (*F*(2,36) = 1.16, *P* = 0.32). The post hoc Newman-Keuls test revealed that CGS 21680 dose-dependently and significantly (*p* < 0.05) reduced the number of active lever presses without any changes in the number of inactive lever presses (Fig. 6, left panel).

Similarly, two-way ANOVA for treatment × lever interaction demonstrated a significant effect of CGS 21680 (1–2.5 ng/side) on the combination of the cue with the subthreshold cocaine (2.5 mg/kg, i.p.) dose (F(2.36) = 3.44, *P* = 0.04). The post hoc Newman-Keuls test revealed that CGS 21680 significantly (*p* < 0.001) attenuated the number of active lever presses without any changes in the number of inactive lever presses (Fig. 6, right panel).

### Intra-PFc effects of the A_2A_ receptor agonist on cocaine seeking behavior

Two-way ANOVA for treatment × lever interaction did not indicate (*F*(2,36) = 1.45, *P* = 0.25) a significant effect of CGS 21680 at a dose of 2.5 ng/side on cocaine (10 mg/kg, i.p.) (*F*(2,36) = 1.45, *P* = 0.25) or on the cue with the subthreshold cocaine (2.5 mg/kg, i.p.) dose (*F*(1,24) = 0.63, *P* = 0.44) effects (Fig. 7).

### Locomotor activity

**Fig. 8:**
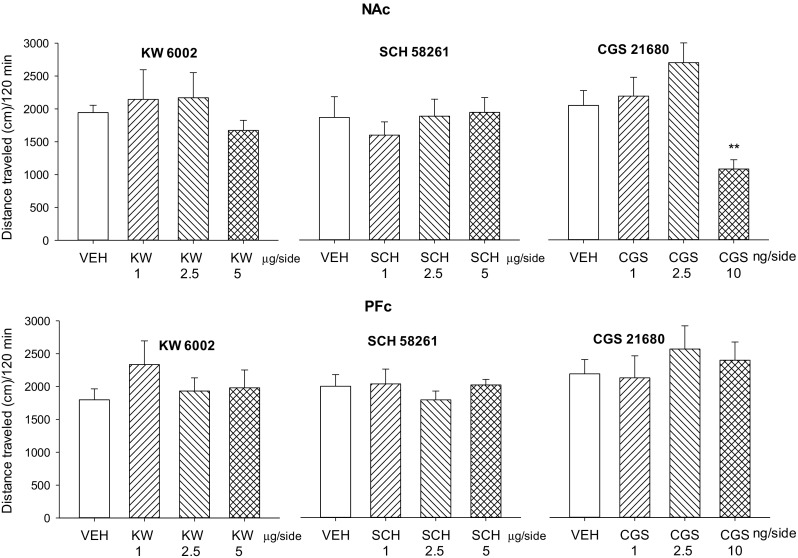
Intra-NAc and intra-PFc effects of the A_2A_ receptor antagonists KW 6002 (KW; 1–5 μg/side), SCH 58261 (SCH; 1–5 μg/side), and the A_2A_ receptor agonist CGS 21680 (CGS; 1–10 ng/side) on the locomotor activity (cm) in rats. **p < 0.01 vs vehicle (VEH) (Dunnett’s test). *N* = 7–8 rats/group

Neither intra-NAc (*F*(3,24) = 0.92, *P* = 0.44) nor intra-PFc (*F*(3,24) = 0.78, *P* = 0.52) injections of KW 6002 (1–2.5 μg/side) influenced locomotor activity of rats (Fig. 8).

Similarly, intra-NAc (*F*(3,24) = 0.37, *P* = 0.77) or intra-PFc (*F*(3,24) = 0.437, *P* = 0.73) injections of SCH 58261 (1–2.5 μg/side) did not alter locomotor behavior of rats (Fig. 8).

However, intra-NAc (*F*(3,27) = 6.56, *P* < 0.001), but not intra-PFc (*F*(3,27) = 0.479, *P* = 0.69), injections of CGS 21680 changed locomotor activity in rats. Post hoc Dunnet’s test revealed that CGS 21680 (10 ng/side) reduced rats’ locomotor activity (Fig. 8).

## Discussion

It was demonstrated that A_2A_ receptors located in the NAc shell, but not in the infralimbic PFc, seem to be engaged in the cocaine rewarding and seeking behaviors in rats. Local administration of the A_2A_ receptor agonist CGS 21680 into the NAc was found to weaken cocaine self-administration. However, A_2A_ receptor antagonists KW 6002 and SCH 58261 given locally into the NAs did not affect cocaine reward. These results supported a role for the agonist induced rapid activation of the A_2A_ receptor in the NAc shell to reduce cocaine reinforcement.

The results of this study strongly support our previous study and the results of other authors that systemic administration of an A_2A_ receptor agonist inhibits both the rewarding and motivational effects of cocaine under fixed and progressive ratio schedules of reinforcement (Knapp et al. 2001; Ruiz-Medina et al. 2011; Soria et al. 2006; Wydra et al. 2015a). Despite the fact that CGS 21680 given peripherally or locally to the NAc or to the dorsal striatum does impair the motor activity in rodents (Barraco et al. 1993; Filip et al. 2006; Hauber and Munkle 1997; Poleszak and Malec 2002), the inhibitory effects of this A_2A_ receptor agonist on cocaine self-administration could not be explained by sedation and a reduction in locomotion. Thus, intra-NAc CGS 21680 (1–2.5 ng/side) had neither effects on the higher dose used for cocaine (0.5 mg/kg/infusion) self-administration nor did it alter the number of inactive lever presses or horizontal locomotor activity. In this paper, local administration of A_2A_ receptor antagonists did not influence cocaine self-administration which supported previous results obtained with systemic administration of these drugs (Wydra et al. 2015a).

Furthermore, we show that A_2A_ receptors located in the NAc affected cocaine seeking behavior. Thus, the A_2A_ receptor antagonist SCH 58261, but not KW 6002, given alone into NAc induced reinstatement of cocaine seeking behavior. The mechanism for the above discrepancy between the two A_2A_ receptor antagonists toward cocaine seeking—also seen after their systemic administration (Wydra et al. 2015b)—is unknown. It may be that SCH 58261 vs KW 6002 blocks the A_2A_ receptor protomers of multiple A_2A_ heteroreceptor complexes and A_2A_ homoreceptor complexes in the reward (D_1_ receptor rich) and antireward (D_2_ receptor rich) neurons with different potencies. The A_2A_-D_2_, A_2A_-D_3_, A_1_-A_2A_, and A_2A_-mGlu5 heteroreceptor complexes may have a special role in modulating the reward and antireward circuitries. They may have different affinities for the A_2A_ receptor antagonists employed which can explain the differential effects observed on cocaine reward and seeking with A_2A_ receptor antagonists (Ciruela et al. 2006; Fuxe et al. 2008; Orru et al. 2011b; O’Neill et al. 2015).

In line with our previous study with systemic administration of A_2A_ receptor antagonists (Wydra et al. 2015b), combination of intra-NAc shell injections of KW 6002 (2.5 and 5 μg/side) or SCH 58261 (5 μg/side) with subthreshold dose of cocaine (2.5 mg/kg, i.p.) induced some additional effect on the number of active lever presses during reinstatement tests. Likewise, O'Neill et al. (2012) showed cocaine-mimicking and additive effects of the intra-NAc core administration of the A_2A_ selective receptor antagonist MSX-3 in combination with cocaine. It should be pointed that other authors show some addictive potential of A_2A_ receptor antagonists since the drug substitutes for cocaine in baboons (Weerts and Griffiths 2003) or produces conditioned place preference in rats.

In contrast to the above antagonists, the intra-NAc shell administrated A_2A_ receptor agonist CGS 21680 (5 ng/side) brought down cocaine-induced seeking behavior evoked by a priming dose of cocaine (10 mg/kg, i.p.) and was also a very effective blocker to the cocaine seeking reinstatement evoked by combination of conditional stimulus + subthreshold dose of cocaine (2.5 mg/kg, i.p.). In fact, in rats with intracranially implanted guide cannulae, it is difficult, if ever, to reinstate cocaine seeking evoked by the conditional stimulus under FR 5 schedule of reinforcement (Acosta et al. 2008, this paper). Supporting the present findings, our (Wydra et al. 2015a) and other authors (Bachtell and Self 2009) studies with systemic CGS 21680 administration demonstrated that this agonist dose dependently reduced cocaine- or cue-induced reinstatement to cocaine seeking. More recently, O'Neill et al. (2012) found that pretreatment with the same A_2A_ agonist administered into the NAc core (with the injection volume 0.5–1 μl/side) dose dependently blunted the cocaine- and cue (under FR1 schedule)-induced reinstatement of cocaine seeking behavior, what together with our present finding (much smaller injection volume of 0.2 μl/side) illustrate that stimulation of A_2A_ receptors localized to both parts of the NAc may control over cocaine seeking. Interestingly, the previous studies with systemic and intra-NAc drug injections indicate that A_2A_ receptors alter D_2_ receptor signaling as CGS 21680 reduced quinpirole-induced reinstatement (Bachtell and Self 2009; O'Neill et al. 2012; Wydra et al. 2015a, b), possibly through antagonistic allosteric A_2A_-D_2_ receptor interactions. The above functional interaction has been raised on tasks involving effort-related processes (Mingote et al. 2008; Pardo et al. 2012), in motivational disruptions of mother-infant interactions (Pereira et al. 2011) or in excessive ethanol drinking (Nam et al. 2013). The latter hypothesis needs, however, to be verify with the local injection of pharmacological tools or with the recently available A_2A_ transmembrane peptide that disrupts the A_2A_-D_2_ heteroreceptor complexes (Borroto-Escuela et al. 2018).

As previously shown, A_2A_ receptor stimulation after high doses and systemic or intra-NAc agonist administration reduced lever pressing for food or sucrose (Font et al. 2008; Wydra et al. 2015a, b), while such reduction was not reported for the minimally effective dose of intra-NAc core CGS 21680 (2.5 ng/side) (O'Neill et al. 2012). On the other hand, intra-NAc MSX-3 reduced food intake and delayed intake onset in food-deprived rats (Nagel et al. 2003) and other studies also strongly indicate brain A_2A_ receptors as the target to control the exertion of effort in motivational behaviors (Correa et al. 2016; Mingote et al. 2008; Pardo et al. 2012). Furthermore, with using optogenetic activation of A_2A_ receptors and satiety-based instrumental training, Li et al. (2016) defined the dorsomedial striatum A_2A_ receptor signaling in relation to the time of the reward and in control of instrumental learning. Whether the observed in this paper modulatory effects of A_2A_ receptor ligands were specific to cocaine reward and seeking behaviors, it remains to be defined in further studies.

A_2A_ receptors are found mostly in striatal regions; however, different populations of A_2A_ receptors in different brain regions bi-directionally control cocaine actions (Shen et al. 2008, 2013). In fact, A_2A_ knockout mice having deficits in A_2A_ receptors in the forebrain (i.e., cerebral cortex, hippocampus, and striatum) or only in the striatum after cocaine treatment provided evidence that widespread forebrain knockout of A_2A_ receptor reduced cocaine-induced locomotion while striatal-specific knockout of A_2A_ enhanced the effects of cocaine (Shen et al. 2008, 2013). To complete our pharmacological analysis, the future studies should include genetic research with using neuron salience strategy, i.e., neuron selective in vivo knockdown to eliminated functional accumbal A_2A_ receptors by rapid siRNA (Nakajima et al. 2012) or CRISPR interference Cas 9 technique for transcriptional repression (Larson et al. 2013). Additionally, the overexpression of A_2A_ receptors by Tet-on/off strategy—if available—will help complete the research on A_2A_ receptors and cocaine addiction.

In the current paper, we studied if local stimulation or blockade of A_2A_ receptor in the infralimbic PFc might affect cocaine self-administration and seeking behavior of rats. The finding that A_2A_ receptor ligands in the infralimbic PFc did not change cocaine self-administration and seeking (as well as rats’ locomotion) means they are not involved in controlling cocaine reward and seeking, but this fact does not eliminate the significance of A_2A_ receptors in different brain regions such as the prelimbic PFc or hippocampus in cocaine behaviors as was found with using genetic tools (Shen et al. 2008, 2013). Interestingly, another genetic report indicates different contribution of A_2A_ receptors to fine-tune information processing in neuronal networks. In fact, the deletion of neuronal A_2A_ receptors is precognitive, while the deletion of astrocytic A_2A_ receptors enhances behavioral impairments (Matos et al. 2015). The astrocytic A_2A_ receptor function is linked with the density of NR2B subunits of the glutamatergic NMDA receptors (Matos et al. 2015), and the latter receptors are upregulated during cocaine withdrawal with extinction training in rats (Pomierny-Chamiolo et al. 2015), what further requires determining the specificity of A_2A_ receptors not only in brain areas but also in cell type and cell compartment.

The current findings reveal the effects of selective A_2A_ receptors stimulation or blockade in the NAc shell and infralimbic PFc on behavioral effects of cocaine. The inhibitory effects of the A_2A_ receptor agonist CGS 21680 on cocaine reward are associated through the activation of A_2A_ receptors in the NAc shell.
